# Demonstration of Logic Operations in High-Performance RRAM Crossbar Array Fabricated by Atomic Layer Deposition Technique

**DOI:** 10.1186/s11671-016-1807-9

**Published:** 2017-01-13

**Authors:** Runze Han, Peng Huang, Yudi Zhao, Zhe Chen, Lifeng Liu, Xiaoyan Liu, Jinfeng Kang

**Affiliations:** Institute of Microelectronics, Peking University, Beijing, 100871 China

**Keywords:** RRAM, Crossbar, Atomic layer deposition (ALD), Logic operation

## Abstract

In this paper, resistive random access memory (RRAM)-based crossbar arrays with the cell structure of Pt/[AlO_*y*_/HfO_*x*_]_*m*_/TiN were fabricated by using atomic layer deposition (ALD) technique. The RRAM devices in the arrays show excellent performances such as good uniformity and high reliability. Based on the fabricated RRAM array, a complete set of basic logic operations including NOR and XNOR were successfully demonstrated.

## Background

Resistive random access memory (RRAM) is regarded as one of the most promising candidates for next generation non-volatile memory due to its advantages such as low cost, fast write/read speed, low-energy consumption, and easy 3D integration [1–4]. In addition to the most common application as memory, RRAM can also be applied to artificial neural network [5], mixed signal computing [6], and logic operation [7–10]. To perform logic operations, the combinations of a few devices in a crossbar array are needed [10]. This characteristic makes it suitable for highly compact crossbar arrays with a 4F^2^ cell area (F is the minimum feature size) [11]. During logic computing process, each cell in a RRAM crossbar array can be used as input, output, assistance, and memory at different stages [8, 10]. This computing in memory ability of the RRAM crossbar array will greatly reduce the time and energy consumption in the data shuttling process between the processing unit and memory [12], thus making RRAM crossbar arrays appealing for developing beyond traditional von Neumann computing architectures [8–10, 13, 14].

However, implementation of logic operations in the RRAM crossbar array also brings challenges for RRAM devices such as reducing device variability, improving device yield, enlarging switching endurance etc. [10, 15, 16]. Former works demonstrated that HfO_*x*_ thin films with embedded Al layers will lead to better device uniformity [17, 18]. In this work, we use ALD technique to deposit a uniform and ultrathin AlO_*y*_/HfO_*x*_ stacked resistive layer ([AlO_*y*_/HfO_*x*_]_*m*_). The fabricated RRAM crossbar array with structure of Pt/[AlO_*y*_/HfO_*x*_]_*m*_/TiN shows excellent device performances such as large resistance window (>80), uniform switching voltage, high switching endurance (>10^7^ cycles), high-disturbance immunity (>10^9^ cycles), and good retention (>10^5^ s at 150 °C). Based on the fabricated RRAM crossbar array, a complete set of basic logic operations including NOR and XNOR were successfully demonstrated.

## Methods

**Fig. 1:**
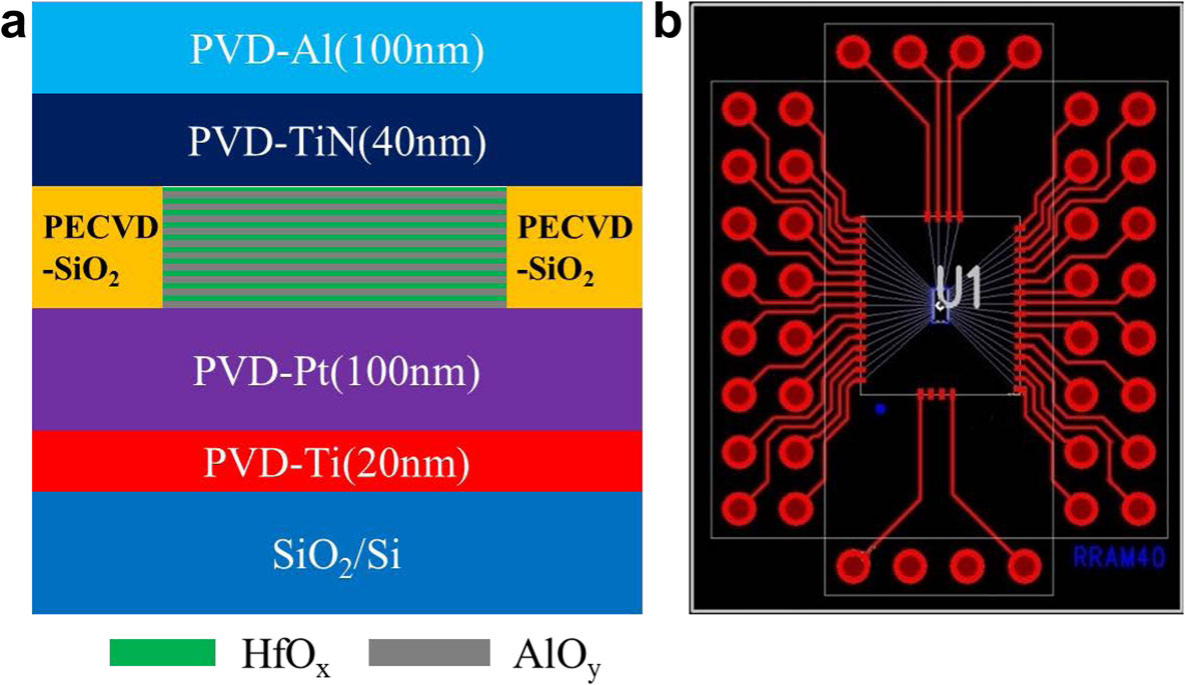
**a** The fabricated RRAM device structure. **b** The packaged RRAM crossbar array

The structure of a single device in the fabricated RRAM crossbar array is shown in Fig. 1a. The sputtered Ti/Pt layers were used for adhesion and bottom electrode, respectively. AlO_*y*_ and HfO_*x*_ were deposited layer by layer using ALD (Picosun R-200, Masala, Finland) technique at 300 °C. H_2_O and trimethylaluminum (TMA)/tetrakis[ethylmethylamino]hafnium (TEMAH) were used as precursors, respectively. This process was repeated for 10 times, so the final composition of the resistive layer is [AlO_*y*_/HfO_*x*_]_*m*_ (*m* = 10). The total thickness of AlO_*y*_ and HfO_*x*_ layers are 3 and 2 nm, respectively. The PECVD-deposited SiO_2_ layer was utilized to isolate resistive layers of different devices. Forty-nanometer TiN top electrode was then sputtered, followed by 100-nm Al contact layer sputtered on TiN. Except of the ALD process for the resistive layer deposition, the rest of the sample fabrication was reported in detail in Ref. [19]. Finally, the fabricated 16 × 2 RRAM crossbar array was packaged by using chip on board bonding method. The packaged crossbar array is shown in Fig. 1b. Note in Fig. 1b, the left and right side represent two crossbar arrays. The contact pads on the left/right side are word lines, while the contact pads on the top side are bit lines.

Electrical measurements were performed by Keysight B1500A semiconductor parameter analyzer (Keysight Technologies, Inc., Santa Clara, CA, USA).and Keysight 81160A pulse pattern and function arbitrary noise generator.

## Results and Discussion

### Device Performances

#### Uniformity

**Fig. 2:**
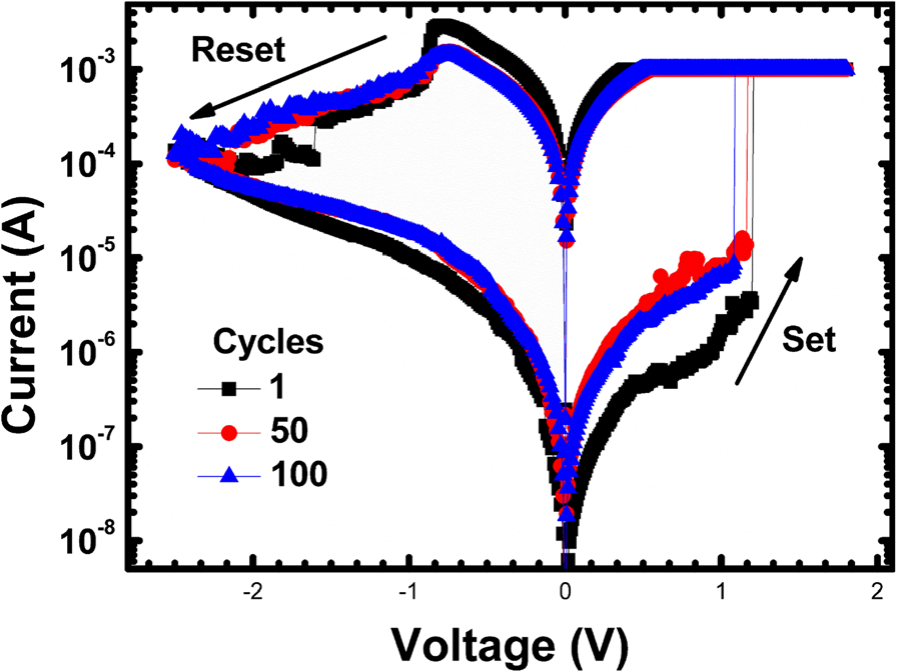
Typical DC I–V curves of the RRAM device in first 100 cycles

**Fig. 3:**
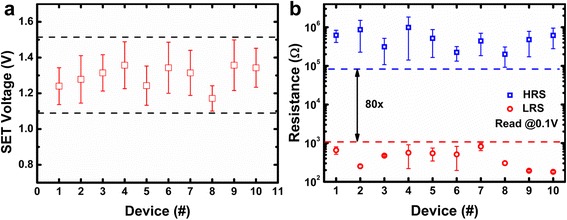
**a** Resistance and **b** set voltage distribution of RRAM devices

Uniformity is a major concern for logic computing. A tight set voltage distribution will make it easier to choose proper amplitudes of reference voltage applied on the input cell and the supply voltage applied on the output cell. Therefore, the misconduct of the output cell will be avoided. The variation exists in the low resistance and high resistance of the RRAM will reduce the resistance window. With a reduced resistance window, the accuracy of the logic operating will be reduced too. So, a uniform resistance distribution is also important for logic operation. The typical DC I–V curves of the RRAM device are shown in Fig. 2. A positive voltage above set voltage (V_SET_) was applied to make the resistance state of RRAM transit from high-resistance state (HRS) to low-resistance state (LRS). During set operation, the current flow through the device is limited to 1 mA to control the filament shape and prevent permanent breakdown. After set transition, a negative voltage lower than reset voltage (V_RESET_) was applied on the device to switch it from LRS to HRS. The good consistency among I–V curves in cycles 1, 50, and 100 reveals excellent cycle-to-cycle switching uniformity of RRAM devices. To investigate the device-to-device variability of fabricated devices, set voltage and resistance distribution of 10 randomly chosen RRAM devices in a crossbar array was tested. To extract the set voltage, a −1.9 V,100 ns voltage pulse was firstly applied on the RRAM device to switch it to a HRS, then a set pulse train with 100-ns width was applied. Set pulse amplitude increases from 0.8 V with a step voltage of 0.05 V; after each step, a read voltage was applied to read its resistance state. When the resistance is lower than a set value (2kΩ), the set pulse amplitude increase stops and the current voltage pulse amplitude was marked as the set voltage. The test result is shown in Fig. 3a. A uniform set voltage distribution range from 1.1 to 1.5 V was measured. According to the measured set voltage distribution, the resistance distribution was tested under 1.6 V,100 ns set pulses and −1.9 V,100 ns reset pulses. The test result suggests that despite cycle-to-cycle and device-to-device variability, RRAM devices still hold a resistance window larger than 80, as is illustrated in Fig. 3b. The excellent uniformity exhibited in these devices is mainly due to the introduction of Al atoms into HfO_*x*_ layers. Because the formation and rupture of the conduction filament consists of oxygen vacancies (V_O_) has been widely recognized as the switching mechanism, while with the diffusion of Al into HfO_x_ layers, the formation energy of oxygen vacancies will be reduced. Therefore, the device uniformity will be improved with more controllable conduction filaments formed steadily along Al atoms [17].

#### Reliability

**Fig. 4:**
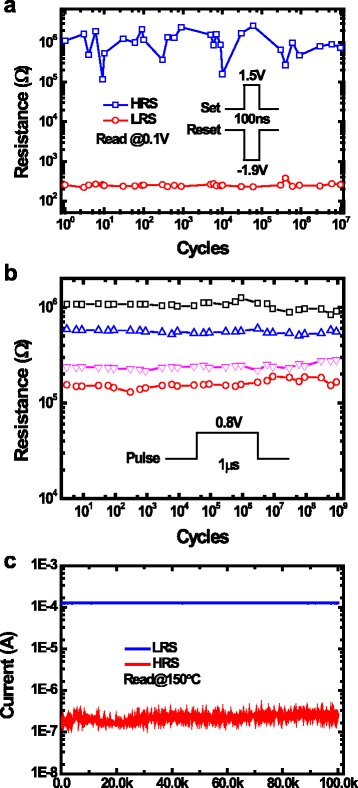
Reliability metrics of RRAM devices. **a** Endurance behavior of RRAM devices. **b** Half-supply voltage disturbance behavior of RRAM devices. **c** Retention behavior

Reliability is another major consideration to build robust computing systems based on RRAM. RRAM devices have to be switched for many times in the computing process, so a good endurance behavior is important. To test the endurance behavior, a 1.5 V,100 ns set voltage pulse was applied on the RRAM device to make it transit from HRS to LRS, then a −1.9 V,100 ns reset pulse was applied to change the resistance state to HRS, this process was repeated for many times. The test result suggests fabricated RRAM devices could switch normally after 10^7^ consecutive switching, which is shown in Fig. 4a. Because the doping of Al atoms in HfO_*x*_ layers will lead to lower formation energy and more controllable filament shape, then the conduction filament will become more recoverable, so the endurance will be enhanced [20]. During logic computing, half supply voltage is applied on the input cell, thus will cause disturbance [10, 21]. Good disturbance immunity ensures the logic state of the input cell will not be changed with applied voltage disturbance. The disturbance behavior is tested under repeated 0.8 V, 1 μs disturbance pulses. Figure 4b shows excellent disturbance immunity of RRAM devices for over 10^9^ disturb cycles with different initial resistance ranges from 0.1 to 1MΩ. To ensure the logic state of the computing cell being correctly sensed, a good retention behavior is needed. Excellent retention behavior for LRS and HRS was measured for up to 10^5^ s at the temperature 150 °C, which is shown in Fig. 4c.

### Demonstration of Logic Operations

**Fig. 5:**
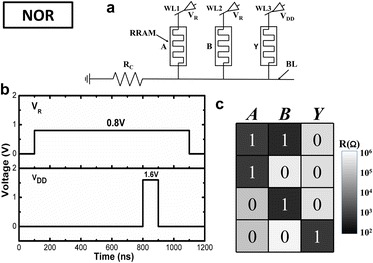
NOR logic operation. **a** Circuit realization. **b** Applied pulse waveforms. **c** The measured resistance truth table

**Fig. 6:**
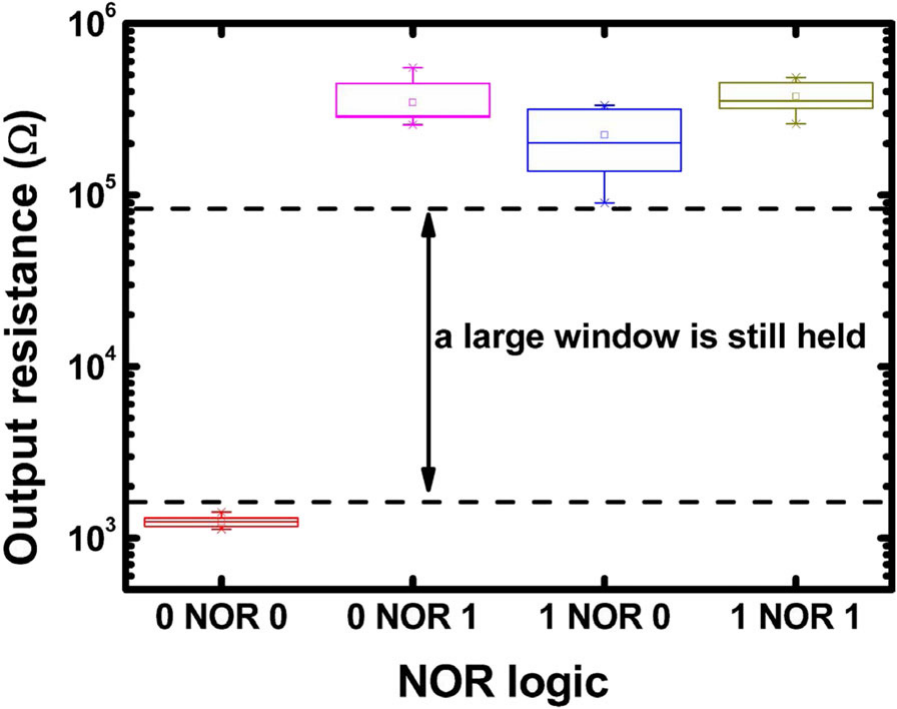
Box plot of the measured NOR logic. Each NOR operation with a certain input combination was repeated for 10 cycles

The set containing NOR suffices as a complete logic set. Any other logic operation can be realized through combinations of NOR logic. Circuit realization of NOR logic operation is shown in Fig. 5a. Three RRAM devices were used with two input cells marked as A and B and one output cell marked as Y. The common bit line is serially connected to GND through a load resistance R_C_. To guarantee the logic operation works correctly, the voltage should fall mainly on the input cells (the cells are all in HRS) or R_C_ (at least one of the cells is in LRS). So, the load resistance R_C_ should be much smaller than HRS while much larger than LRS. The choosing criteria of the R_C_ are illustrated in detail in Ref. [10]. Referring to the measured resistance distribution shown in Fig.3b, R_C_ is set to 22kΩ. Word lines of A and B are connected to reference voltage (V_R_) while the word line of Y is connected to supply voltage (V_DD_). Figure 5b shows the applied pulse waveforms. V_R_ should be smaller than V_SET_ to ensure the input cell state does not change; V_DD_ should be larger than V_SET_ to ensure the output cell could be switched normally from HRS to LRS. Regarding of the SET voltage distribution, the amplitudes of V_DD_ and V_R_ are set to 1.6 and 0.8 V, respectively. The narrower V_DD_ is applied in the middle of V_R_ to make sure the initial electrical potential of common bit line is determined by the input cells, thus avoiding error operation of output device. Before the operation, the output cell is switched to HRS (logical 0). If input cells A and B are all in HRS, then the parallel resistance is still high. Because R_C_ is much smaller than V_R_, the electrical potential of common bit line will remain close to ground due to voltage dividing. Therefore, the voltage drop across Y is approximate to supply voltage V_DD_, which is higher than set voltage, leading to SET operation and transition of the resistance state of Y to LRS (logical 1). While for other cases, the parallel resistance of A and B is low, so the electrical potential of common bit line will be raised, thus reducing the voltage drop across output cell Y, resulting in the maintenance of the resistance state of Y. The measured resistance truth table of the logic operation is shown in Fig. 5c. From Fig. 5c, we can see that only when A and B are both in the HRS, then the output cell Y is switched to LRS, which demonstrates the NOR logic operation works correctly. To demonstrate NOR logic computation is repeatable, we repeated this logic operation for 10 cycles. The statistical box plot is shown in Fig. 6. Although resistance variations exist in different resistance states, the statistical result suggests that the logic computing still functions correctly with a sufficiently large logical window.

**Fig. 7:**
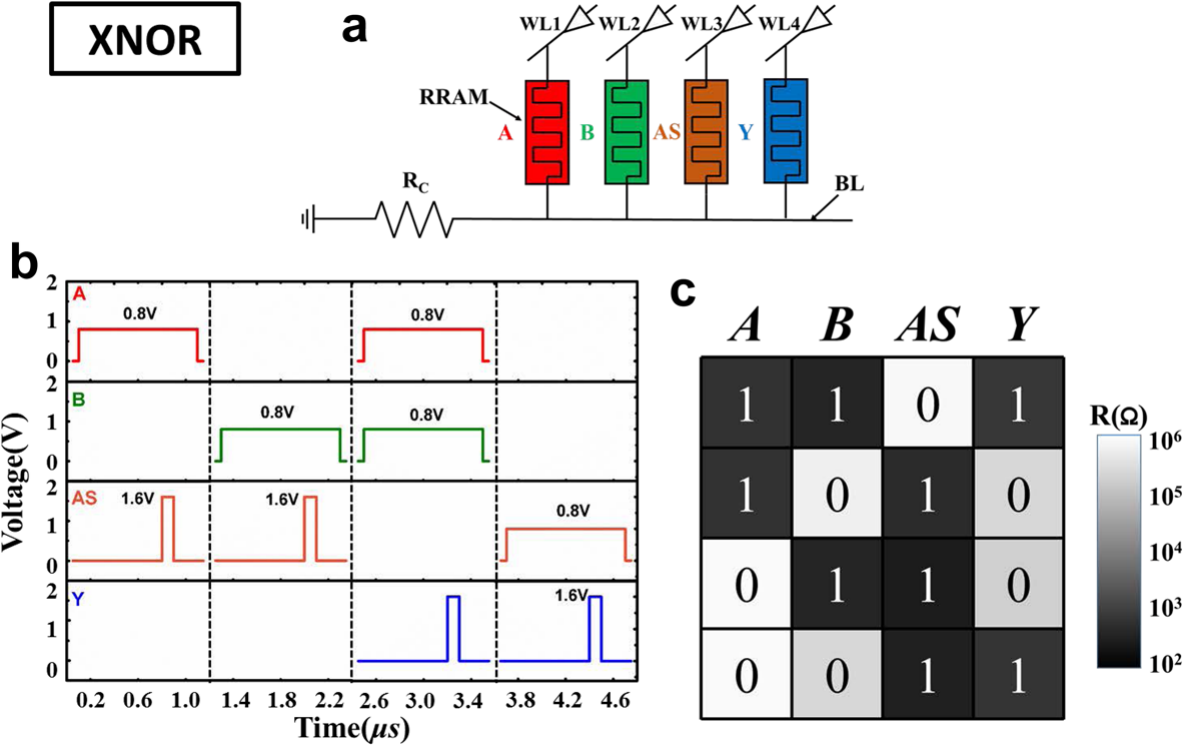
XNOR logic operation. **a** Circuit realization. **b** Applied pulse waveforms. **c** The measured resistance truth table

XNOR logic is widely used in constructing full adder and parity check. Circuit realization of XNOR logic operation is shown in Fig. 7a. Compared with circuit realization of NOR logic, an additional assistant cell marked as AS is used. Figure 7b shows the applied pulse-train waveforms. Before the operation, AS and the output cell *Y* are switched to HRS. The XNOR logic operation is realized by four steps. In the first step, V_R_ is applied to A and V_DD_ is applied to AS. This is a basic imply logic, so after step 1, AS = *Ā*. In the second step, V_R_ is applied to *B* and V_DD_ is applied to AS. Because AS has already been turned to *Ā* in the first step, so after step 2, AS is equal to $\overline{A}\kern0.5em +\kern0.5em \overline{B}$. In the third step, V_R_ is applied to *A* and *B*; V_DD_ is applied to *Y*; this is a NOR logic operation, so the third step realized $Y\kern0.5em =\kern0.5em \overline{A+B}$. In the fourth step, V_R_ is applied to *A* and V_DD_ is applied to *Y*. Because *Y* has already turned to $\overline{A+B}$ in the third step, so after step 4, *Y* is equal to $\overline{\overline{A}+\overline{B}}+\overline{A+B}\kern0.5em =\kern0.5em \mathrm{AB}+\overline{\mathrm{A}}\overline{\mathrm{B}}$. Therefore, after the complete pulse train was applied, XNOR logic operation was achieved. The measured resistance truth table is shown in Fig. 7c, only when *A* and *B* are in the same resistance state, then the output cell is turned to LRS, which demonstrated the XNOR logic functions correctly.

## Conclusions

Logic operations including NOR and XNOR were successfully demonstrated in the ALD-fabricated RRAM crossbar arrays. Excellent performances such as large resistance window, uniform switching, and high reliability were achieved in the cells of this ALD-fabricated RRAM crossbar arrays, which provides opportunity for logic operations in the RRAM crossbar arrays.

## Abbreviations

ALD: Atomic layer deposition
HRS: High-resistance state
LRS: Low-resistance state
PCB: Printed circuit board
RRAM: Resistive random access memory
V_DD_: Supply voltage
V_R_: Reference voltage
V_RESET_: RESET voltage
V_SET_: SET voltage
